# Mediator Subunits MED16, MED14, and MED2 Are Required for Activation of ABRE-Dependent Transcription in Arabidopsis

**DOI:** 10.3389/fpls.2021.649720

**Published:** 2021-03-11

**Authors:** Morgan Lee, Anna Dominguez-Ferreras, Ewon Kaliyadasa, Wei-Jie Huang, Edna Antony, Tracey Stevenson, Silke Lehmann, Patrick Schäfer, Marc R. Knight, Vardis Ntoukakis, Heather Knight

**Affiliations:** ^1^Department of Biosciences, Durham University, Durham, United Kingdom; ^2^School of Life Sciences, University of Warwick, Coventry, United Kingdom; ^3^Warwick Integrative Synthetic Biology Centre, University of Warwick, Coventry, United Kingdom; ^4^Institute of Molecular Botany, Ulm University, Ulm, Germany

**Keywords:** mediator complex, ABA, MED16, MED14, MED2, transcription, protoplast, ABRE

## Abstract

The Mediator complex controls transcription of most eukaryotic genes with individual subunits required for the control of particular gene regulons in response to various perturbations. In this study, we reveal the roles of the plant Mediator subunits MED16, MED14, and MED2 in regulating transcription in response to the phytohormone abscisic acid (ABA) and we determine which *cis* elements are under their control. Using synthetic promoter reporters we established an effective system for testing relationships between subunits and specific *cis-*acting motifs in protoplasts. Our results demonstrate that MED16, MED14, and MED2 are required for the full transcriptional activation by ABA of promoters containing both the ABRE (ABA-responsive element) and DRE (drought-responsive element). Using synthetic promoter motif concatamers, we showed that ABA-responsive activation of the ABRE but not the DRE motif was dependent on these three Mediator subunits. Furthermore, the three subunits were required for the control of water loss from leaves but played no role in ABA-dependent growth inhibition, highlighting specificity in their functions. Our results identify new roles for three Mediator subunits, provide a direct demonstration of their function and highlight that our experimental approach can be utilized to identify the function of subunits of plant transcriptional regulators.

## Introduction

Transcriptional regulators are commonly assembled in multi-subunit protein complexes that regulate varied transcriptional responses. Individual subunits within each complex facilitate the expression of specific gene regulons in response to appropriate stimuli, contributing to specificity in gene activation (Martinez, 2002). However, identifying the function of individual subunits can be challenging. An exemplar of this complexity is the Mediator transcriptional coactivator complex. The complex is conserved across all eukaryotes, where it is a key requirement for the expression of most protein-coding genes by forming a bridge between condition-specific transcription factors and RNA polymerase II (Pol II) (Conaway and Conaway, 2011). The Mediator complex is required for constitutive as well as inducible gene expression (Ansari et al., 2009) and consists of approximately 25–35 subunits, depending on the species (Bjorklund and Gustafsson, 2005). Subunits make up four functional submodules; the head, middle, tail, and kinase domains (Dotson et al., 2000). The head takes part in multiple associations with Pol II and is linked to the tail by the middle; the tail is considered to be the main site of transcription factor (TF) binding and as such, is the least conserved part of the complex (Maji et al., 2019), with tail subunits showing the most inter-species divergence (Bourbon, 2008; Conaway and Conaway, 2011). The kinase module binds reversibly to the main body of the complex, depending on external stimuli (Jeronimo and Robert, 2017). Individual subunits of the kinase module also facilitate specific transcriptional events, for instance the expression of developmental and stress-responsive genes in plants (Gillmor et al., 2010; Ng et al., 2013). Following its original discovery in yeast three decades ago (Flanagan et al., 1991), the Mediator complex was also isolated from plants (Backstrom et al., 2007). The homology of the complex subunits between species is weak at the sequence level although the structural homology is remarkably well-conserved (Cai et al., 2009).

The plant Mediator complex is currently estimated to consist of 33 subunits (Yang et al., 2016; Zhai and Li, 2019) and has been shown to control gene expression during development and in response to abiotic and biotic stresses (Mathur et al., 2011; Yang et al., 2016). Different plant Mediator complex subunits play roles in the expression of some but not all genes and this is thought to contribute to specificity in gene activation (Hemsley et al., 2014; Dolan and Chapple, 2018). We previously identified the Mediator subunit 16 (MED16) as the protein product of *SENSITIVE–TO-FREEZING6* (*SFR6*), a gene with a role in plant acclimation to freezing tolerance (Knight et al., 2009). MED16, predicted to be part of the tail submodule, is required for the cold-inducible expression of genes controlled by the CBF [C-repeat binding factor TFs (Gilmour et al., 2004)] (Knight et al., 1999, 2009; Hemsley et al., 2014). However, MED16’s role extends beyond cold signaling, influencing the transcriptional response to darkness (Hemsley et al., 2014), UV (Wathugala et al., 2012), pathogens (Wathugala et al., 2012; Zhang et al., 2013), osmotic stress (Boyce et al., 2003), heat stress (Crawford et al., 2020), phosphate starvation (Raya-Gonzalez et al., 2020), and iron deficiency (Yang et al., 2014; Zhang et al., 2014). MED16 also regulates cell wall biosynthesis (Sorek et al., 2015), cell growth (Liu et al., 2019) and the response to circadian signals (Knight et al., 2008). Despite this seemingly ubiquitous role, MED16 controls only specific regulons that use particular *cis*/*trans-*acting factor combinations. In the case of cold-responsive genes, only those activated *via* the C-repeat [CRT/DRE; drought responsive element (Yamaguchi-Shinozaki and Shinozaki, 1994)] or the evening element [EE (Mikkelsen and Thomashow, 2009)] fall within the control of MED16 (Hemsley et al., 2014).

Previous work showed that MED16 is also required for abscisic acid (ABA)-independent osmotic stress-responsive expression *via* the DRE promoter motif (Boyce et al., 2003), whose activation requires the action of the drought-responsive element binding-2 (DREB2) transcription factors (Liu et al., 1998; Nakashima et al., 2009). However, this study was not able to reveal whether MED16’s role in osmotic-stress responsive expression extends to an influence on the ABA-dependent pathway. The phytohormone ABA is an essential “stress hormone” and plays a range of roles in integrating a variety of stress signals, notably drought, and coordinating downstream responses (Vishwakarma et al., 2017; Chen et al., 2020). Accumulation of ABA during the onset of drought triggers rapid responses, including guard cell closure (Munemasa et al., 2015), as well as slower responses, many of which are mediated through changes in the expression of thousands of ABA-responsive genes (Song et al., 2016). ABA-dependent transcriptional responses require the actions of ABA-responsive TFs including the AREB bZIP family of TFs (ABA-responsive element binding factors or ABFs; ABRE-binding factors) (Guiltinan et al., 1990). The AREBs regulate ABA-mediated transcriptional responses by specifically binding to the ABRE-binding motif PyACGTGG/TC in the promoters of their target genes (Choi et al., 2000; Uno et al., 2000).

Whether MED16 is required in the control of drought-responsive genes *via* the ABA-dependent pathway has not previously been elucidated. MED18, MED19a, and MED25 are, to date, the only plant Mediator subunits with demonstrated roles in the response to ABA (Chen et al., 2012; Lai et al., 2014; Li et al., 2018). This study sets out to show whether MED16 and neighboring subunits MED2 and MED14 play a role in ABA-dependent transcriptional activation. Using synthetic promoter reporter constructs we demonstrate a reliance on these three Mediator subunits for ABA-dependent expression controlled specifically by the ABRE motif. We also show that the three subunits play a role in specific ABA-dependent physiological responses.

## Materials and Methods

### Chemicals

Unless otherwise stated, all chemicals were obtained from Sigma (Poole, United Kingdom) or Fisher Scientific (Loughborough, United Kingdom).

### Plant Material and Growth Conditions

The Arabidopsis EMS mutant *sfr6-1* (described here as “*med16-1*”) and T-DNA insertion lines *med2-1* (SALK_023845) and *med14-2* (SAIL_373_C07) have been described previously (Knight et al., 2009; Hemsley et al., 2014) and were grown alongside Columbia-0 wild type plants (Col-0). Linkage between these mutations and Mediator function have been demonstrated previously with the use of multiple alleles (Hemsley et al., 2014). Plants were grown on 1 × Murashige and Skoog and 0.8% agar in nine-cm diameter Petri dishes placed in 16:8 h (light:dark) cycle at 20°C in a Percival CU-36L5 growth chamber (CLF PlantClimatics, Wertingen, Germany) except when transferred to vertical media for root length assays. Root length measurements were made on seedlings grown for 7 days on MS medium as above and then transferred to square vertical plates containing 1X MS and 1.2% agar supplemented with 0 or 20 μM ABA for 5 days before photographing and measurement using image J software^1^. Plants used for leaf water loss assays were grown on peat plugs as described before (Hemsley et al., 2014) under 12:12 (light:dark) conditions to promote vegetative growth. Seedlings grown as described above were transferred to plugs after 8 days on agar plates. Plants were placed in a water-saturated environment (well-watered trays in sealed plastic bags) 16 h prior to leaf excision to ensure stomata were open. Leaves were weighed individually, immediately following excision and at hourly intervals afterward. Between measurements leaves were kept in individual polycarbonate weigh boats at 20°C on the lab bench.

### ABA Treatments and Gene Expression Measurement in Seedlings

For gene expression experiments, 8–10-day-old seedlings were transferred to 6-well culture dishes in the light, containing either 100 μM ABA or the appropriate ethanol control treatment (0.1% ethanol) with tissue harvested promptly after 6 h. RNA was extracted and quantitative RT-PCR (qRT-PCR) analysis of gene expression was performed exactly as described previously (Hemsley et al., 2014) using primers listed in Supplementary Table 1. *PEX4* was used as the housekeeping gene for normalization. In each biological repeat experiment three technical replicate wells were used for each sample/primer pair combination. Three independent biological replicate experiments were conducted on three different occasions and the data pooled.

### Statistical Analysis

A linear mixed effects (LME) model (Kuznetsova et al., 2016) was computed using R software (R-Core-Team, 2016), with genotype and treatment (i.e., ABA) specified as fixed terms, and experiment specified as a random effect. qRT-PCR results were analyzed by performing a two-way ANOVA on delta C_t_ values with an interaction term specified between genotype and ABA. Pairwise analysis was carried out using a least-square means (LSM) comparison (Lenth, 2016) to assess significant differences in expression of each gene between genotype and/or treatment. The same analysis was applied to leaf water loss data.

### Cloning ABF4

The AREB2 (ABF4) coding sequence was amplified from full-length Arabidopsis cDNA using the primers: 5′-CACCATGGGAACTCACATCAATTT-3′ (forward) and 5′- CATTAACCGGACCATGGTGA-3′ (reverse). The coding sequence was then cloned into pENTR-D-TOPO entry vector (Invitrogen, Carlsbad, CA, United States) using an Invitrogen pENTR^TM^ Directional TOPO^®^ Cloning kit, as per manufacturer’s instructions. The coding region was then transferred into the Agrobacterium binary destination vector pK7WGF2 (Karimi et al., 2002) by Gateway LR recombination using Gateway^TM^ LR Clonase^TM^ II Enzyme mix (Invitrogen, Carlsbad, CA, United States).

### Promoter Analysis

The 5′ UTR plus 500 bp upstream sequence of *KIN2*, *RAB18*, and *LTI78* were searched for motifs on either the sense or antisense strand corresponding to the ABRE (ACGTGGC, ACGTGTC, GACACGT, or GCCACGT) and DRE (ACCGAC, GCCGAC, GTCGGT, or GTCGGC) (Supplementary Figure 1).

### Reporter Luciferase Constructs

Native promoter sequences of *RAB18* and *LTI78* coupled to a luciferase coding sequence were constructed previously using the plasmid *pFRK1:LUC* (Lehmann et al., 2020). The transfection control plasmid was *pAtUBQ10:GUS*. Minimal promoter sequences (−46 minimal promoter) coupled to four tandem repeat copies of either the DRE or ABRE motif were designed using Snapgene^2^, synthesized by Integrated DNA Technologies (Leuven, Belgium) and cloned *via* Gibson assembly (Gibson, 2009) into the *Nco*I site of pDH51 upstream of a LUC+ (codon-optimized luciferase) coding region (Whalley et al., 2011). A minimal promoter lacking any concatamer sequence was used as the control.

### Protoplast Isolation and Transfection

Protoplasts were prepared from 4–5 week-old Col-0 or mutant plants grown in soil in a controlled environment with 12 h light at 22°C and 12 h dark at 20°C (60% relative humidity). The isolation of mesophyll protoplasts was performed as described previously (Wu et al., 2009). The isolated protoplasts were transfected, and the expression of the reporter constructs was analyzed according to Lehmann et al. (2020).

## Results

### MED16, MED14, and MED2 Are Required for ABA-Responsive Expression

Previously we showed that the Mediator complex subunit MED16 is required for cold-responsive *COR* gene activation by the CBF (DREB1) TFs, which bind the CRT/DRE motif (Yamaguchi-Shinozaki and Shinozaki, 1994). Many cold-responsive (*COR)* genes are also drought-responsive, and the expression of the same *COR* genes was compromised in *sfr6* (from now on referred to as *med16*) mutant plants responding to osmotic stress (Boyce et al., 2003). However, whilst analysis of the transcriptomic data identified a number of DRE-containing genes as misregulated in *med16* (Boyce et al., 2003), not all *med16*-misregulated genes contained a DRE motif, suggesting that one or more additional *cis*-acting element(s) might be under the control of MED16.

**FIGURE 1:**
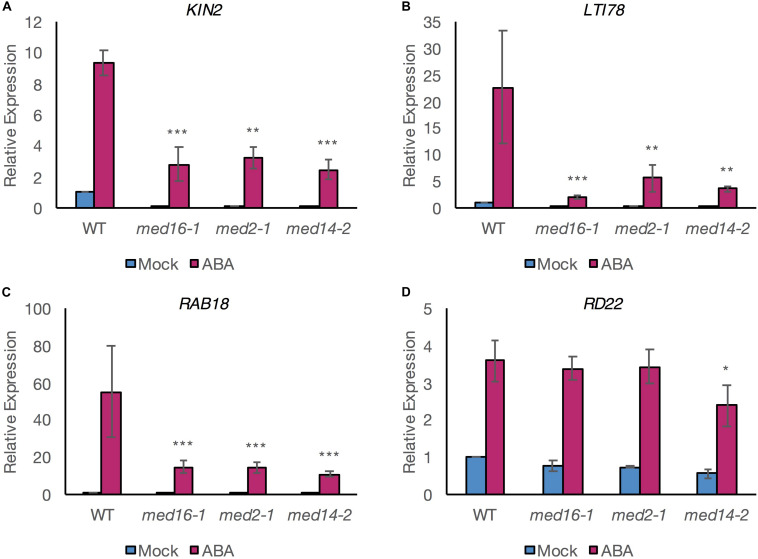
ABA-responsive expression of specific ABRE- and DRE-containing genes is compromised in mutants of the Mediator subunits MED2, MED14, and MED16. qRT-PCR analysis for relative expression levels of *KIN2* **(A)**, *LTI78* **(B)**, *RAB18* **(C)** (all containing at least one copy of both DRE and ABRE motifs in their promoter; Supplementary Figure 1), and *RD22* **(D)** (containing a copy of neither motif). Relative expression was assessed in 8–10-day-old seedlings of Col-0 (WT) and Mediator subunit mutants exposed to 100 μM ABA (pink bars) or mock treatment (blue bars) for 6 h. Relative expression levels to the *PEX4* housekeeping gene and normalized to mock-treated WT seedlings are shown. Data shown are mean values of three independent biological replicate experiments with ± 1 SE. For each gene, a linear mixed effects model was computed and statistical significance was analyzed using least square mean (LSM) analysis. Asterisks denote LSM significance compared to the ABA-treated WT plants: ***P < 0.001, **P < 0.01, and *P < 0.05.

To investigate the possibility that MED16 could control drought-responsive expression *via* the ABA-dependent pathway leading to activation of the ABRE motif, we quantified the expression of well-studied ABA- and drought-inducible genes, in *med16* mutants following exogenous application of ABA. We expanded our analysis to the tail subunit mutants *med2-1* and *med14-2*, as they also show reduced cold-inducible *COR* gene expression like *med16* (Hemsley et al., 2014). ABA treatment induced the expression of the three *COR* genes *KIN2*, *LTI78* (*RD29A*), and *RAB18* in all genotypes but the expression levels were significantly lower in *med16-1*, med2*-1*, and *med14-2* mutants than in wild type plants (Figures 1A–C).

This indicated that the MED16, MED2, and MED14 subunits are all required for at least part of the ABA-mediated transcriptional responses. The upstream sequences of these three genes contain ABRE and DRE motifs (Supplementary Figure 1). In contrast, expression of *RD22*, which is dependent on MYC/MYB TFs for upregulation (Abe et al., 1997) and whose promoter contains neither an ABRE nor a DRE motif was induced by ABA to a similar level in *med16-1* and *med2-1* mutants and wild type (Figure 1D). This suggested that MED16 and MED2 do not control the expression of all ABA-responsive genes but might specifically regulate genes containing an ABRE and/or a DRE motif. A small but significant reduction in the expression of *RD22* in response to ABA was observed in *med14-2* (Figure 1D), suggesting an additional role for this subunit in the regulation of genes *via* MYC and MYB TFs (Abe et al., 1997; Shinozaki et al., 2003).

Our results showed that ABA-induced transcript levels of three ABRE-containing genes were reduced in mutants lacking MED16, MED2, or MED14. However, these experiments measured total transcript levels, which are dependent on additional factors such as transcript stability. Therefore, we addressed the issue of transcriptional activation directly in further experiments.

### MED16, MED14, and MED2 Are Required for Activation of ABA-Responsive Promoters

**FIGURE 2:**
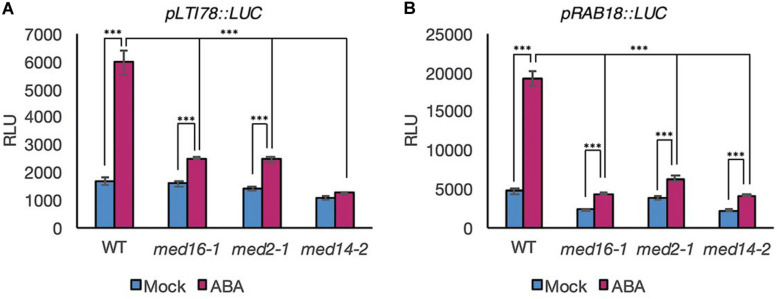
Luciferase activity driven by the promoter of specific ABRE- and DRE-containing genes in response to ABA is lower in mutants of the Mediator subunits MED2, MED14, and MED16 compared to the WT. Luciferase activity measured in protoplasts transformed with a *pLTI78:LUC* **(A)**, or *pRAB18:LUC* **(B)** construct and exposed to 30 μM ABA (pink bars) or mock treatment (blue bars) for 3 h. Data shown are mean values of four repeats. Error bars represent ± one SE. Asterisks denote significant difference between the indicated treatments or genotypes: ***P < 0.001. One representative experiment out of three is presented.

To ascertain whether MED16, MED2, and MED14 are required for ABA-responsive transcriptional activation of drought gene promoters, we adopted a protoplast transformation system (Lehmann et al., 2020). This allowed transient expression of promoter reporter constructs in wild type and mutant backgrounds to be measured and facilitated a direct assessment of promoter activation, eliminating any possible confounding effects of differential transcript stability. Protoplasts of all four genotypes were transformed with reporter constructs in which the native promoter of *LTI78* and *RAB18* fused to a coding sequence for luciferase (Lehmann et al., 2020). Consistent with whole seedling data, ABA treatment increased the activation of the *LTI78* and *RAB18* promoters in Col-0 to significant levels (*p* < 0.001 for both gene constructs, ANOVA) but the levels of expression in the three Mediator subunit mutants were significantly lower than those observed in wild type (Figure 2).

The data indicated that the three Mediator subunits affect transcriptional activation of the *LTI78* and *RAB18* promoters rather than exerting an effect through altering transcript stability. The similar behavior of both genes in protoplasts and seedlings also demonstrated that protoplasts were a suitable experimental system for further investigation of the transcriptional response to ABA.

### MED16, MED14, and MED2 Are Specific Requirements for Activation of Expression *via* the ABRE Motif

The necessity for the three Mediator subunits in the transcriptional response to ABA could suggest they control ABRE activation, however, whilst the promoters of *LTI78* and *RAB18*, (like *KIN2*), contain at least one copy of an ABRE motif, they also each contain one or more copies of the DRE motif (Supplementary Figure 1). Therefore, the DRE motif could act as a coupling element for the ABRE motif (Narusaka et al., 2003).

**FIGURE 3:**
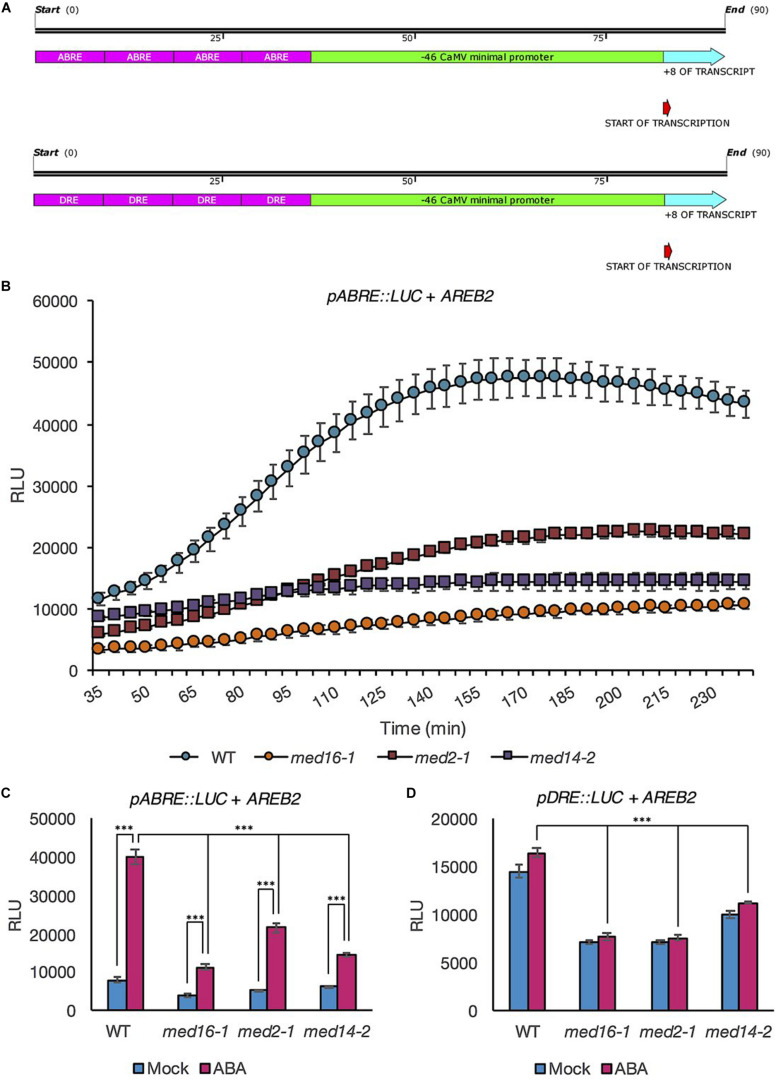
Luciferase activity driven by the ABRE and DRE promoter motifs in response to ABA is lower in mutants of the Mediator subunits MED2, MED14, and MED16 compared to the wild type. Maps showing constructs in which the coding sequence for LUC+ was fused to –46 bp minimal *CaMV* promoter driven by four copies of the ABRE motif and minimal promoter driven by four copies of the DRE motif **(A)**. Protoplasts of wild type (Col-0; blue circles), *med16-1* (orange circles), *med2-1* (red squares), and *med14-2* (purple squares) plants were transformed with the *pABRE:LUC* construct and *AREB2*. They were then treated with ABA (10 μM) and the luciferase activity was recorded for 4 h. Data shown are mean values of four repeats. Error bars represent ± one SE. One representative experiment out of three is presented **(B)**. Luciferase activity measured in protoplasts transformed with *AREB2* and a *pABRE:LUC* **(C)** or *pDRE:LUC* **(D)** construct and exposed to 10 μM ABA (pink bars) or mock treatment (blue bars) for 3 h. Data shown are mean values of four repeats. Error bars represent ± one SE. Asterisks denote significant difference between the indicated treatments or genotypes: ***P < 0.001. One representative experiment out of three is presented.

Our protoplast system allowed us to directly quantify the contribution of MED subunits to ABA-responsive activation of the ABRE by making synthetic reporter constructs in which four copies of either the ABRE or DRE were fused to a minimal promoter (−46 bp of the CaMV promoter) (Ali and Kim, 2019) coupled to the LUC (Figure 3A). Activation of the synthetic reporter constructs was tested following ABA treatment in protoplasts co-transformed with AREB2 (ABF4) in order to further enhance expression levels (Fujita et al., 2005). AREB2 has been shown experimentally to bind to the ABRE and is involved in the response of vegetative tissues to ABA (Kang et al., 2002; Gao et al., 2016). Protoplasts of all four genotypes showed increased ABRE-driven induced expression upon ABA treatment (Figure 3B). However, expression activation was slower and reached a lower maximum level in all three Mediator subunits mutants compared with wild type. ABRE activation levels measured after 3 h of treatment were significantly higher in ABA-treated than mock-treated protoplasts (*p* < 0.001 for each genotype, ANOVA). Importantly, the activation of the ABRE by ABA was clearly reduced in the Mediator subunit mutants (Figure 3C). The induction was lowest in *med16-1* with the mildest effect observed in *med2-1* (Figure 3C). The addition of ABA to protoplasts co-transfected with AREB2 had no effect on luciferase expression driven by the minimal promoter in the absence of a concatamer (Supplementary Figure 2); as expected, neither did expression of the DRE concatamer change in response to ABA (Figure 3D). However, expression levels of the DRE reporter in the three mutants were significantly lower than in the wild type (Figure 3D; *p* < 0.001 for each genotype, ANOVA), reflecting our previous observations that these subunits affect uninduced *COR* gene expression levels (Hemsley et al., 2014). Collectively these data showed that complete activation of the ABRE motif requires MED16, MED14, and MED2.

**FIGURE 4:**
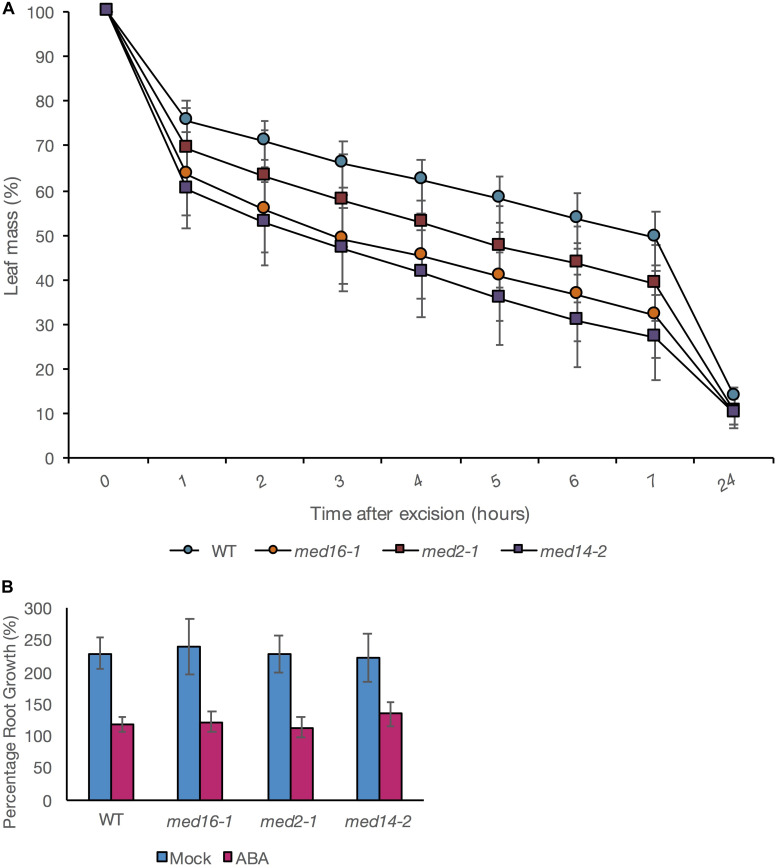
Effect of Mediator mutations on ABA-mediated inhibition of root growth and control of leaf water loss. **(A)** Leaf mass expressed as a percentage of mass at the point of excision and after 1, 2, 3, 4, 5, 6, 7, or 24 h. The average of four independent biological replicate experiments is presented. In each experiment; each datapoint is derived from seven individual leaves, each from a different plant. Error bars show standard ± 1 standard error. **(B)** Relative root lengths of seedlings 5 days after transfer to vertical plates containing 0 (blue bars) or 20 μM ABA (pink bars). Data shown are mean values of three biological replicates with eight root measurements per genotype per experiment and error bars indicating ± 1 SE. Results are presented as a% of original length before transfer. Any differences in the effect of ABA on root length between the WT and mutant plants was NS (*p* > 0.05).

To test whether the mutants simply exhibited a global reduction in sensitivity to ABA, we examined two ABA-dependent responses in intact wild type and mutant plants. We assessed leaf water loss (a measure of water lost *via* stomata, regulated by ABA) following leaf excision to evaluate the capability of mutant plants to respond to dehydration stress (Hopper et al., 2014). Leaf water loss was significantly greater in *med16* and *med14* mutants than in wild type at all time points measured from 1 to 7 h after leaf excision. *med2-1* exhibited greater water loss than wild type though differences were significant only at 7 h after excision (Figure 4A). In contrast, ABA-mediated inhibition of root growth was not impaired in any of the mutants (Figure 4B). An ANOVA performed on a linear mixed effects (LME) model of the relative change in root growth after ABA treatment showed all differences to NS when compared with WT (*p* = 0.6538, 0.2224, and 0.7676 for *med16*, *med14*, and *med2*, respectively. This indicated that the three Mediator subunits play a role in a specific subset of ABA-depended responses, most likely due to interactions with specific AREBs. Thus, our approach was able to dissect the specific role of the Mediator subunits 16, 14, and 2 in implementing specific transcriptional responses to ABA through the ABRE motif.

## Discussion

The Mediator complex is a multi-subunit transcriptional regulatory complex, conserved across eukaryotes. The plant Mediator complex consists of 33 subunits, four of which are plant-specific (Zhai and Li, 2019). Whilst there is much published evidence to identify the genes that are under the control of each Mediator subunit, there has been less research linking particular *cis*-*trans* acting factor interaction events with control by particular Mediator subunits (Mao et al., 2019). Possible subunit-TF relationships have been inferred indirectly, using bioinformatic analysis of the promoters of misregulated genes in Mediator subunits mutants. We previously used this approach to reveal the association between MED16 and genes containing the DRE motif (Hemsley et al., 2014) and recently it has been successfully employed in a study that links different Mediator subunits with promoter motifs that include CAMTA and WRKY binding sites (Crawford et al., 2020).

However, with this approach is not always possible to differentiate between particular *cis*-*trans* acting motifs. For example, our previous work showed that in addition to exhibiting compromised cold-inducible gene expression and reduced freezing tolerance (Knight et al., 1999), the mutant of the MED16 subunit was also compromised in the induction of the same *COR* genes in response to an artificial osmotic stress stimulus (Boyce et al., 2003). At the time of that study, we attributed this to the fact that the affected genes were those regulated *via* the CRT/DRE motif (Yamaguchi-Shinozaki and Shinozaki, 1993) through the actions of the DREB1 (CBF) and DREB2 TFs in response to cold and osmotic stress respectively (Liu et al., 1998). Whether this was due to an effect on ABA-dependent as well as ABA-independent activation could not be elucidated until we conducted the present study. To address this point, we treated seedlings with ABA to distinguish ABA-dependent expression from osmotically-induced expression *via* the ABA-independent route, which is reliant on the DREB2 TFs (Yoshida et al., 2014). Our results showed that ABA-responsive expression of the *COR* genes *KIN2, LTI78*, and *RAB18* was compromised in mutants of MED16, MED14, and MED2.

To directly test the dependency of *cis*-*trans* elements activation on plant Mediator subunits we express synthetic reporter constructs in protoplasts. Our observation that the ABA-induced transcriptional differences between the WT and mutants were qualitatively very similar in seedlings and protoplasts convinced us that protoplasts provide a reliable system for testing the activation of specific motifs. Like many other *COR* genes (Baker et al., 1994; Narusaka et al., 2003), the tested genes contained putative DRE as well as ABRE motifs in their promoter (Supplementary Figure 1), so we could not exclude the possibility that the three Mediator subunits exerted their effect indirectly *via* the DRE acting as a coupling element. However, published evidence suggests that the putative DRE motif in *RAB18* is not functional, as it does not respond to overexpression of constitutively active DREB2A (Sakuma et al., 2006). Furthermore, *RAB18* expression is unchanged by overexpression of CBF2 (DREB1B) (Vogel et al., 2005) or in the *cbfs* triple mutant (Jia et al., 2016), making this unlikely to be a functional DRE/CRT motif. Therefore, the reduced activation of *RAB18* in the Mediator subunit mutants suggested that a mechanism other than the DREB2-DRE interaction was involved. This points to the possibility that the three Mediator subunits were regulating ABA-dependent *COR* gene expression *via* a motif other than the DRE, most likely the ABRE. The suggestion that the Mediator mutants were affecting activation *via* the ABRE was further supported by the fact that ABA-responsive expression of *RD22*, a gene containing neither ABRE nor DRE motifs in its promoter, was barely affected in the mutants. A small but significant reduction of *RD22* expression was detected in *med14-2*, but this was not surprising given the pivotal role of this subunit and may have been a consequence of the fact that MED14 is likely to attach the whole of the tail submodule to the complex (Cevher et al., 2014; Maji et al., 2019).

To directly address the dependency of each motif on Mediator subunits, we produced synthetic concatamers of either the ABRE or DRE motif fused to a minimal promoter. Codon-optimized LUC+ was used as expression levels were anticipated to be lower when driven by isolated *cis*-elements coupled to a minimal promoter than they are with full promoters. The concatamers designed were identical to those described previously by Whalley et al. (2011), with four copies of each motif in tandem to increase the chances of expression (Rushton et al., 2002). We also reduced the length of the minimal CaMV promoter sequence to 46 bp to limit the possibility of promoter activation through unintended motifs. In these experiments, we co-transfected the protoplasts with a construct for AREB2 in order to further enhance expression levels (Fujita et al., 2005). All three mutants showed significantly reduced ABRE-motif-driven activation in response to ABA treatment. This indicates that all three subunits play a significant role in activating the ABRE motif. Our results demonstrate a new role for three Mediator subunits in the response to ABA and provide clear evidence that they are required specifically for activation of expression *via* the ABRE motif.

Our data show a strong reliance on the three Mediator subunits for the transcriptional activation of the ABRE-containing synthetic promoters and the control of leaf water loss, a process that is strongly influenced by the effect of ABA on stomatal closure. This demonstrated the functional significance of our findings. However, we could not assume that every ABRE activation event is dependent on these subunits. Sensitivity of root growth to ABA was unaltered in any of the three mutants compared with wild type plants, confirming that a further level of specificity is involved.

Previous work is consistent with the idea that different aspects of the response to ABA are controlled by different subunit members of the plant Mediator complex. MED25 interacts with ABI5 and MYC TFs and effects negative regulation (Chen et al., 2012) whilst MED19A interacts with ABI5 and *med19a* mutants show reduced transcription of ABRE-containing genes and reduced sensitivity of root growth and germination to ABA (Li et al., 2018). MED18 interacts with ABI4 and germination of *med18* shows reduced sensitivity to ABA (Lai et al., 2014). It appears, therefore, that different aspects of the transcriptional response to ABA are governed by a particular set of Mediator subunits that each interact with specific ABA-responsive TFs to control a subset of ABA-dependent genes and downstream processes. Our previous work showed that MED16 acts with MED2 and MED14 to control CBF-regulated *COR* gene expression but that dark-responsive expression requires MED16 acting in conjunction with MED5 but not MED2 (Hemsley et al., 2014).

Thus, our results are consistent with a model in which the involvement of the Mediator subunits in transcriptional responses depends on the stimulus, promoter architectures and the interactions between subunits and TFs. Furthermore, the approach we present will aid in further unraveling the function of subunits of other plant transcriptional regulators.

## Data Availability Statement

The original contributions presented in the study are included in the article/Supplementary Material, further inquiries can be directed to the corresponding author/s.

## Author Contributions

VN and HK devised the experiments and wrote the manuscript. PS contributed to experimental design. ML and AD-F performed the experiments and analyzed the data. MRK analyzed data and designed constructs and TS and EA made constructs. EK, W-JH, EA, and SL performed experiments. All authors contributed to the article and approved the submitted version.

## Conflict of Interest

The authors declare that the research was conducted in the absence of any commercial or financial relationships that could be construed as a potential conflict of interest.

## Footnotes

1 imagej.net

2 www.snapgene.com
