# Supplementary material for: Mediator Subunits MED16, MED14, and MED2 Are Required for Activation of ABRE-Dependent Transcription in Arabidopsis
Source: Front Plant Sci. 2021 Mar 11;12:649720. doi: 10.3389/fpls.2021.649720 (PMC7991908; doi:10.3389/fpls.2021.649720)
Supplement: Supplementary file 1 [file Data_Sheet_1.docx]

Supplementary Material: Lee et al.

## Supplementary Figures

**Supplementary Figure 1**.

5’UTR + 500 bp of upstream sequence for *KIN2*, *LTI78* and *RAB18* showing positions of ABRE and DRE motifs. Consensus used for ABRE is ACGTGG/TC (marked in pink); consensus for DRE used is A/GCCGAC (marked in yellow).

***KIN2*** ATTCAACTTGTCAACAAATATACAACTTATATGACATGTCTGGCTCCAAACTGGATTTGAGTAGAAAGTTACTAATTCACAAAAACTAATTTATATACACATTTTCAATTTTTATTTTATAAATCAAAGAAAAAAAACATGAAAATACGGGAGGTTCGGCAAACACAACATTTAACTTGCCAAACGTAGCATCTAACTATCCCACCTTCTACAAGGCACCATTTTTTTTTCAATAATAAAGTTTTTTTTCTTTAATTTCTTCGCCAAGAAGAGCACGAAATGTTTGCCAAACGCATATGCAACAACCCCACGTTACATATTTCTATTTGTAGCTATAGAGCAAGCTATATTGTTAAAAACTAAAAAGAAAATCTTTACTATAACATATAGATAGAGGATTCGAGATATCTTGAAAGACTCAACTTAATAAATAAAGTCGAAAAGAAAACACGGAGGCGAGAGGACCACACACTCGCACAGAAAGAGTCTCATATCCTCTATAACAAATTGATAAACTAAACTAAAACGACACGTGATGTCTTGATCAGCCAATAAAAAGCTACCGACATAAGGCAAAAATGATCGTACCATTAAACGTAATCCACGTGGTTTCAGATTACACGTGGCACCACACAAGTATCTCCATTTGGCCTATAAATATAAACCCTTAAGCCCACATATCTTCTCAATCCATCACAAACAAAACACACATCAAAAACGATTTTACAAGAAAAAAATATCTGAAAA

***LTI78*** GGAGGTAAACATTTTCTTCTATTTTTTCATATTTTCAGGATAAATTATTGTAAAAGTTTACAAGATTTCCATTTGACTAGTGTAAATGAGGAATATTCTCTAGTAAGATCATTATTTCATCTACTTCTTTTATCTTCTACCAGTAGAGGAATAAACAATATTTAGCTCCTTTGTAAATACAAATTAATTTTCGTTCTTGACATCATTCAATTTTAATTTTACGTATAAAATAAAAGATCATACCTATTAGAACGATTAAGGAGAAATACAATTCGAATGAGAAGGATGTGCCGTTTGTTATAATAAACAGCCACACGACGTAAACGTAAAATGACCACATGATGGGCCAATAGACATGGACCGACTACTAATAATAGTAAGTTACATTTTAGGATGGAATAAATATCATACCGACATCAGTTTGAAAGAAAAGGGAAAAAAAGAAAAAATAAATAAAAGATATACTACCGACATGAGTTCCAAAAAGCAAAAAAAAAGATCAAGCCGACACAGACACGCGTAGAGAGCAAAATGACTTTGACGTCACACCACGAAAACAGACGCTTCATACGTGTCCCTTTATCTCTCTCAGTCTCTCTATAAACTTAGTGAGACCCTCCTCTGTTTTACTCACAAATATGCAAACTAGAAAACAATCATCAGGAATAAAGGGTTTGATTACTTCTATTGGAAAGAAAAAAATCTTTGGAAA

***RAB18*** ATGAAAGCTCAAAAATAAATTCAATCCGATTCCTTTAGTGATATCAGAAGTTCATTTTAAATACGAACACGTATGGCGAAACACCACGCCGACATTTTCTGCTGCTGCCACGCGTCACTTTCCAAATATTGATTCATTAAACTAATAGTTGATCCATATCCGAAACCGGACTATAAAACTATCTTCAATGCGTTAACGAATCTTCATCGATCAAACTCATCAAAGTCTAATATCACAAAGAAAGAGTTTTTTTAACTAGCTTAGCTCAAAGTGTTTGCTTAAGACAAGAAGAACCAGATTCGAGAGGGTCGACTTCACCGTATCCGGATCCATCTCTTTACCAACCAACTAATCCAACTCAGAAAATTTTAAAATCTCAATCAAAAATCCCTCTAAGATAGCCAGAGAAGAGATTGTAAACAAGGATTTGAAATCTGGTGCAGAGAGGAGAAACTCCCCGACAATGAACACCAACGATCTAAACGCGGCGTTTGGTAAAAGTTGAGTAAATTTTGTTAGGGCTTAGTTTTAGTCCATGGGCTAATTAGTAAGTGATTTACGGCCCACACATGAGCCCAAATGTTTCAGACCCAGCCAAGTTTCTTCAAATTCACCCAATCAACGACGATGTACGTGTGTATGAAAATCATTAACACGACGCATCGCTTTCGAGGAGGAGCATTACGTGTCCTGTTAGCTACGATAATGTTAGTACCGCCACAAAGAAAAGGATAGATATTTTGCTTTCCAGCACCCTGTCATGGGATTGATATGAACACGTACTTGGTATCGAC

**Supplementary Figure 2.**

Effect of ABA and AREB2 co-transfection on activation of a -46 bp minimal *CaMV* promoter coupled to LUC+.


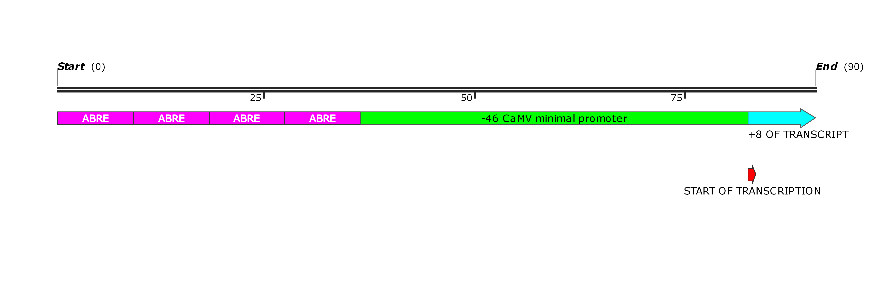


**
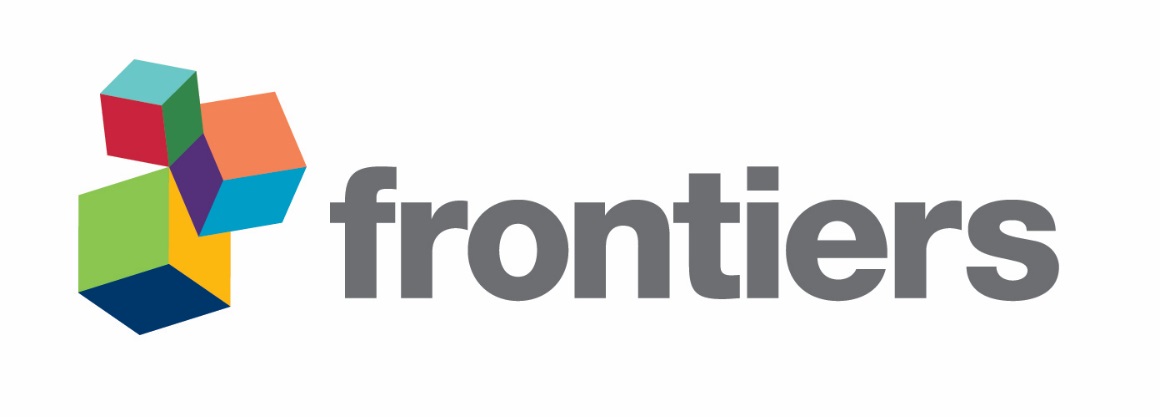
**
